# Epidemiology of mucormycosis in COVID-19 patients in northwest Iran: *Rhizopus arrhizus* as the predominant species

**DOI:** 10.22034/cmm.2025.345248.1591

**Published:** 2025-06-01

**Authors:** Kambiz Diba, Rahim Nejadrahim, Kosar Jafari, Marzieh Safari, Rasool Jafari, Narges Aslani

**Affiliations:** 1 Department of Parasitology and Mycology, School of Medicine, Urmia University of Medical Sciences, Urmia, Iran; 2 Department of Infectious Diseases and Dermatology, School of Medicine Taleghani Hospital, Urmia University of Medical Sciences, Urmia, Iran; 3 Department of Medical Laboratory Sciences, Urmia Branch, Islamic Azad University, Urmia, Iran

**Keywords:** COVID-19, Co-infection, Diabetes mellitus, Iran, Mucormycosis, Mucorales

## Abstract

**Background and Purpose:**

The current study aimed to assess the demographic features, clinical characteristics, species diversity, and contributing factors among patients with severe acute respiratory syndrome coronavirus-2 pneumonia-associated mucormycosis in northwestern Iran.

**Materials and Methods:**

This cross-sectional descriptive study was performed on patients who tested positive for COVID-19 via reverse-transcriptase-polymerase chain reaction and were suspected of having invasive fungal infection. Mucormycosis was confirmed by histopathology of biopsy samples and species identification was performed using morphological and internal transcribed spacer-rDNA sequencing methods.

**Results:**

Mucormycosis was observed in 63 COVID-19 patients. Mean age of patients was 56.65±14.49 years (range of 22-85 years) and 63.5% of the involved patients were male. The most common involvement site of patients with mucormycosis was the sinus (63.5%). Among all participants, 84% of patients (n=53) had received intravenous dexamethasone, and 25.4% of them had diabetes mellitus. All patients with proven invasive mucormycosis received intravenous amphotericin B. In total, 21 (33%) positive cultures were identified and *Rhizopus arrhizus* was the main causative agent.

**Conclusion:**

Awareness among physicians should be raised that corticosteroid therapy not only causes dysfunction of the immune system but may also lead to the development of this neglected mycosis through corticosteroid-induced diabetes in vulnerable patients.

## Introduction

In early 2020, the world faced a formidable pandemic disease caused by a single-stranded ribonucleic acid virus named severe acute respiratory syndrome coronavirus-2 (SARS-CoV-2), and its ravaging complications still persist [ 1 ]. Although the direct harmful effects of COVID-19 on the respiratory tract are important, the numerous predisposing therapies and various underlying conditions during the COVID-19 pandemic are the culprits behind the increasing incidence of secondary infections [ 2 ]. These risk factors are probably due to the outcome of COVID-19-related treatments, such as the use of high doses of systemic corticosteroids, which cause immunological suppression and induced hyperglycemia or prophylactic and empirical therapeutic to prevent subsequent opportunistic infections, especially in an intensive care unit (ICU) admitted patients [ 3 , 4 ].

Since management and detection of co-infections and super-infections in patients infected with SARS-CoV-2 is difficult, this leads to increased fatality [ 2 ]. Among opportunistic agents causing secondary infections in cases of COVID-19, invasive fungal infections have been increasingly disclosed with high mortality rates. Although rarely reported as an opportunistic infection in otherwise healthy individuals, COVID-19-associated mucormycosis (CAM) is a deadly fungal infection as it is difficult to diagnose and treat [ 5 , 6 ].

Based on previous investigations, different geographic areas, ecology, and environment are co-factors related to CAM and urgently need to be investigated by local epidemiology. Based on statistics, the frequency of the fungi belonging to the order Mucorales causing mucormycosis ranged from 0.005 to 1.7 per million people globally, while it was approximately 80 times higher in India [ 2 , 6 , 7 ].

Following the COVID-19 report in Iran, cases of CAM have been observed in different parts of the country during the fifth wave of COVID-19 [ 2 , 8 ]. To the best of our knowledge, other studies in Iran have never reported the occurrence of CAM in patients who were simultaneously infected with COVID-19 in Northwest Iran before. The present study aimed to assess the demographic, clinical characteristics, and distribution of Mucorales species among patients diagnosed with CAM in Northwest Iran.

## Materials and Methods

### Study design

This cross-sectional descriptive study was conducted on hospitalized patients with a history of COVID-19 who were admitted to Infectious Diseases Hospital, Urmia University of Medical Sciences, Urmia, Iran, from February 2020 to the end of May 2021. Following previous studies, the clinical samples collected from the patients (clinically suspected invasive fungal infection) included nasal and sinus discharge samples, biopsies of nasal septa, oral palates, and debridement of facial and orbital tissues [ 9 ]. The mentioned specimens were referred to the medical mycology laboratories of Urmia University of Medical Sciences. All procedures of this study were conducted in accordance with the Helsinki Declaration and approved by the Ethics Committee of Urmia University of Medical Sciences (IR.UMSU.REC.1402) [ 3 ]. Written informed consent was obtained from all patients participating in this study and their clinical data were legally used for research objects.

### Case definition, data collection

**Figure 1:**
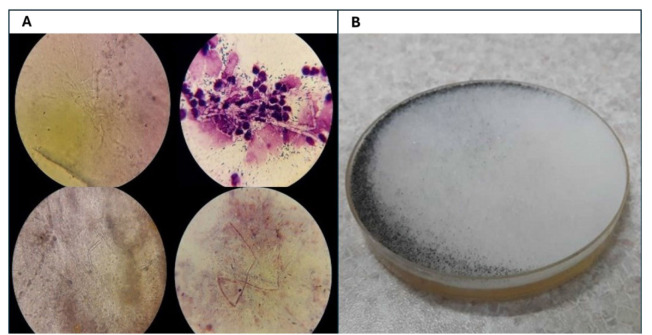
**A.** Pathologic figures of the patients with COVID-19-associated mucormycosis (broad branching non-septate hyphae). **Figure 1B.** Positive culture of *Mucorales* of the biopsy specimens.

Participants were patients whose respiratory specimens (nasopharyngeal or oropharyngeal swab) were verified for SARS-CoV-2 infection by a positive result of reverse-transcriptase-polymerase chain reaction (RT-PCR). This study included concurrent, confirmed mucormycosis patients with histopathologic evidence and demonstration of angioinvasive fungi, characterized by broad irregularly branched aseptate hyphae (Figure 1A), or positive culture of *Mucorales* from biopsy specimens included in this study was done (Figure 1B). The collected para-clinical data included demographic characteristics (age and gender) and the site of clinical specimens.

### Microbiological Definitions

laboratory confirmation of COVID-19 infection was performed using RT-PCR on respiratory samples. The culture from biopsy specimens with positive histopathology assessment as the initial identification of mucormycosis was performed on Sabouraud dextrose agar medium (SDA; Difco, Leeuwarden, The Netherlands) supplemented with chloramphenicol (0.05 mg mL^–1^) at 30 °C for 7 days. The macroscopic and microscopic examinations were conducted on each grown colony of Mucorales from the clinical setting. Subsequently, genomic DNA was extracted from all grown isolates according to the previously described instructions [ 10 ] and DNA was stored at -80 °C before use. For the accurate identification of all strains to the species level, sequencing the internal transcribed spacer (ITS)-rDNA region was performed on amplified fragments of the ITS-rDNA gene using ITS1 and ITS4 primers (ITS-1: 5'- TCC GTA GGT GAA CCT GCG G - 3' and ITS-4: 5'- TCC TCC GCT TAT TGAT TAT GC - 3') as the universal primers [ 11 , 12 ]. Sequencing was carried out on an ABI 3730 automatic sequencer (Applied Biosystems, Foster City, CA). Each sequence data was manually aligned using MEGA 5.05 and searched at the NCBI website (http://www.ncbi.nlm.nih.gov/BLAST/) and the maximum identity (cutoff of ≥97%) was recorded as genus and species names. Moreover, all sequence data were submitted to the NCBI website (http://blast.ncbi.nlm.nih.gov/Blast.cgi).

### Statistical analysis

Statistical analyses of all data were performed in SPSS software (Version 19.0). The obtained results were presented as percentages and median.

## Results

During the study period, 70 SARS-CoV-2 pneumonia patients who were suspected of invasive fungal infection were included in the present investigation. Finally, 63 (90%) patients with laboratory-confirmed COVID-19 and mucormycosis were included in this study. Mean age of the patients was 56.65±14.49 years (range of 22-85 years) and 63.5% of patients were male gender with a higher prevalence in this group. The most common involvement sites of patients with mucormycosis were sinus (63.5%), cerebra (16%), and orbital (11%).

Moreover, DM was the underlying disease in 16 (25.4%) patients.  Regarding gender, 11 (68.8%, ranging from 36 to 85 years old) out of the 16 CAM patients with diabetes mellitus were male. Demographic characteristics of the patients (age and gender) are summarized in Table 1. Among all, 53 patients (84%) had received intravenous dexamethasone (6 mg once daily as the preferred dose in hospitalized patients) for management of COVID-19. Furthermore, among 63 patients with CAM, 8 (9.5%) patients had a severe form of COVID-19 with diffuse lung involvement. In addition, one of the female patients with CAM showed a raised erythrocyte sedimentation rate. The most common admission sites for patients with CAM were the Ear, Nose, and Throat ward with 23 patients (37%), 60.9% of whom were male, the neurosurgery ward with 23 patients (37%), 65.2% of whom were male, and ICU with 12 patients (19%). Generally, the signs and symptoms of infection observed in CAM patients included edema (n=44, 69.9%), facial pain (n=25, 39.7%), acute vision loss (n=10, 15.9 %), and headache (n=3, 4.8%). It should be mentioned that four patients had pulmonary mucormycosis (Table 1).

**Table 1:** Demographic characteristics of 21 COVID-19 patients co-infected with mucormycosis caused by Rhizopus arrhizus.

Case N.	Specimen	Age	Gender	Site	Background	Accession N.	Clinical
1	Edema	52	M	ICU	COVID+, Diabetes, ESR	PQ308986	Sinus involvement
2	Facial pain/edema	41	M	Neurosurgery	COVID+	PQ309680	Sinus involvement
3	Edema	56	M	Neurosurgery	COVID +	PQ310122	Cerebral invasion
4	Edema	53	M	ENT	COVID+ Diabetes	PQ310250	Orbital invasion
5	Edema	66	M	Neurosurgery	COVID+	PQ310356	Cerebral invasion
6	Edema	48	M	Pulmonary	COVID+	PQ326117	Pneumonia
7	Edema/acute vision loss	55	M	Neurosurgery	COVID+	PQ326123	Cerebral invasion
8	Edema	51	M	ENT	COVID+	PQ326124	Sinus involvement
9	Edema	22	M	ENT	COVID+	PQ326126	Sinus involvement
10	Edema	32	M	Neurosurgery	COVID+	PQ326127	Cerebral invasion
11	Edema	41	M	Neurosurgery	COVID+	PQ309060	Cerebral invasion
12	Edema/acute vision loss	32	M	Neurosurgery	COVID+	PQ326121	Sinus involvement Facial invasion
13	Facial pain	53	M	ENT	COVID+	PQ326403	Sinus involvement
14	Facial pain/edema	52	M	ENT	COVID+	PQ309110	Sinus involvement
15	Edema/acute vision loss	76	M	Neurosurgery	COVID+	PQ309532	Sinus involvement
16	Facial pain/edema	67	M	ICU	COVID+	PQ309876	Sinus involvement
17	Facial pain	54	F	ENT	COVID+ Diabetes	PQ309881	Sinus involvement Orbital invasion
18	Edema	23	F	Pulmonary	COVID+	PQ310102	Pneumonia
19	Edema	62	F	Neurosurgery	COVID+	PQ326128	Sinus involvement
20	Headache	64	F	Neurosurgery	COVID+	PQ310228	Sinus involvement Facial invasion
21	Headache	22	F	Infection	COVID+	PQ325937	Sinus involvement

ICU: intensive care unit, ENT: ear, nose and throat, ESR: erythrocyte sedimentation rate

Intravenous amphotericin B (AmB) as the first-line drug for all forms of invasive mucormycosis is prescribed for all patients with proven invasive mucormycosis (IV 5 mg/kg/day 3-6 weeks). Direct histopathology investigation of the sinus discharges and biopsy of tissue samples detected 63 cases of mucormycosis. Out of these 63 cases, 21 (33%) had positive culture results, which were further identified at the species level by sequencing the ITS-rDNA region. Based on the findings, *Rhizopus arrhizus* was the main causative agent, as listed in Table 1.

## Discussion

Certain risk factors, notably, taking high-dose systemic steroids, DM, and immune dysregulation following the recent devastating pandemic caused by SARS-CoV-2, predispose individuals to life-threatening opportunistic infections [ 7 ]. This study investigated COVID-19 patients who had been simultaneously infected with mucormycosis, as an opportunistic fungus, in Northwest Iran. In this study, our assessment of 63 patients with the SARS-CoV-2 Delta variant indicated that R. arrhizus was the main causative agent of mucormycosis.

Based on previous studies, the incidence of invasive candidiasis and aspergillosis as the most common and famous fungal involvements in patients with COVID-19 was higher [ 2 , 13 - 15 ]. Accordingly, based on the meta-analysis by Gioia et al., the diagnosis rates of COVID-19-associated pulmonary aspergillosis as invasive aspergillosis ranged from 2.5% to 47.2% [ 16 ]. Bauer et al. and Zakhem et al. in different multicenter literatures found that the incidence rate of fungemia by *Candida* species during the pandemic was substantially higher in contrast to the pre-pandemic period [ 17 , 18 ]. Nevertheless, the mortality rates of mucormycosis in COVID-19 patients in the previous studies are remarkable and have rates of 14% and higher [ 14 ]. Given the difficulty of correct diagnosis, rapid development, and aggressive, the mortality rate of CAM patients is high. In addition, co-morbidities, especially DM and taking high-dose systemic immunosuppressants (e.g., corticosteroids) put patients at a higher risk of death. The latter risk factor manages and mitigates the hyperinflammatory response during severe COVID-19 and leads to immune imbalance and increased predisposition to opportunistic infections, such as mucormycosis [ 15 , 19 ].

The present study elucidated that 53 patients (84%) had received corticosteroids (i.e., intravenous dexamethasone) for COVID-19 treatment. While in similar studies on COVID-19 patients with mucormycosis, aspergillosis, and oropharyngeal candidiasis, 47%, 40-66%, and 47% of cases had received corticosteroid, respectively [ 3 , 20 , 21 ]. The latest systematic review and meta-analysis performed by Jeong et al. on 851 mucormycosis cases without association with COVID-19 elucidated that corticosteroid usage had a minimal effect on the mortality rate [ 22 ]. Interestingly, the study by Eshraghi et al. observed that the use of systemic corticosteroids during COVID-19 had reduced the odds of mortality but extended the duration of stay in an ICU [ 19 , 23 ]. In the meantime, it seems that ensuing steroid therapy, especially using high-dose steroid treatment, impairs mucosal surface immunity and hyperglycemia-induced. This situation predisposed individuals to secondary opportunistic infections like mucormycosis especially those with DM which lowers the survival rate [ 3 , 24 ]. Based on previous evidence, the important role of DM as the prevailing underlying condition in CAM infection has been confirmed [ 19 ].

The present study revealed that 16 (25.4%) of CAM patients had DM as the traditional underlying condition for mucormycosis. The latest prevalence of this predominant co-morbidity in CAM cases was reported at 77.1% [ 25 ]. Timely diagnosis and appropriate treatment of mucormycosis in COVID-19 patients with DM are life-saving and essential for positive outcomes. Mean age of patients in the current study was 56 years, which is similar to that in a recent meta-analysis that investigated 17 studies (54.6 years) [ 26 ]. According to the same study from Iran, the prevalence of the male gender was higher (63.5 %) in our investigation [ 5 ].

To the best of our knowledge, this is the first molecular identification study performed on COVID-19 patients infected with mucormycosis in northwest Iran. Based on findings of broad irregularly branched aseptate hyphae as hallmarks of mucormycosis infection in tissue samples, the diagnosis was established through histopathology (67 %). In terms of species diversity, R. arrhizus (33 %) was the most common causative agent in the current assessment, similar to a recent molecular study in which R. arrhizus (84.6 %) was the isolated agent in CAM patients. However, *Mucor circinelloides*, *Mucor, Apophysomyces*, and *Lichtheimia* were the species causing mucormycosis in other similar studies [ 2 , 8 , 27 ].

Despite treatment with AmB as an effective and mainstay drug for life-threatening mucormycosis, therapeutic failure due to substantial nephrotoxicity of the mentioned drug and the angioinvasive nature of the Mucorales species may occur and worsen patient outcomes [ 14 ]. Therefore, for successful treatment of severe cases of mucormycosis, empirical usage of liposomal AmB with less nephrotoxicity effect, management of underlying conditions, and surgical debridement of necrotic tissue are necessary. This study highlighted that the awareness of clinicians should be raised about life-treating mucormycosis, especially in diabetic patients or individuals who developed diabetes following long-term corticosteroid therapy.

## Conclusion

Physicians must recognize that corticosteroids not only suppress immunity but can also trigger diabetes, especially in high-risk patients, promoting opportunistic fungal infections like this neglected mycosis. Vigilant glucose monitoring and early suspicion of fungal complications are crucial, even without typical immunosuppression. Improved education, screening, and interventions can reduce infection risks and improve outcomes. Further research should clarify how steroid-induced diabetes fosters fungal growth to guide prevention strategies.
